# Mural Endocarditis and Embolic Pneumonia Due to *Trueperella pyogenes* in an Adult Cow with Ventricular Septal Defect

**DOI:** 10.3390/vetsci8120318

**Published:** 2021-12-09

**Authors:** Domenico Caivano, Maria Chiara Marchesi, Piero Boni, Fabrizio Passamonti, Noemi Venanzi, Elvio Lepri

**Affiliations:** 1 Department of Veterinary Medicine, University of Perugia, Via San Costanzo 4, 06126 Perugia, Italy; fabrizio.passamonti@unipg.it (F.P.); noemi.venanzi@studenti.unipg.it (N.V.); elvio.lepri@unipg.it (E.L.); 2 Veterinary Practice, Via Martiri di Modena 2, 06033 Perugia, Italy; pieroboni@libero.it

**Keywords:** atrial fibrillation, bacterial endocarditis, cattle, echocardiography

## Abstract

Bacterial endocarditis represents one of the most frequently acquired cardiac diseases in adult cattle. Congenital heart diseases as a ventricular septal defect can facilitate bacterial endocarditis as a consequence of turbulent blood flow through the defect, causing damage to the endocardium. We describe a case of mural endocarditis associated with a ventricular septal defect in an eight-year-old female Holstein Friesian cow. The cow’s history revealed that she had presented dysorexia and a sudden decrease of milk production in the last 10 days prior to the examination. On clinical examination, jugular pulses, tachycardia with irregular heart rate and tachypnea with harsh bronchovesicular sounds were evident. Electrocardiographic examination allowed the diagnosis of an atrial fibrillation with high ventricular response rate. Transthoracic echocardiography revealed a large vegetation originating from the endocardium between the tricuspid and pulmonic valves in the right ventricle outflow. On post-mortem examination, a small muscular septal defect under the right coronary cusp of the aortic valve and a mural vegetative endocarditis were found. An abscess in the chondro-costal junction of the third right rib and metastatic pneumonia were also observed. This case report describes a rare consequence of a small ventricular septal defect that had not been previously diagnosed in an adult cow.

## 1. Introduction

Bacterial endocarditis represents one of the most frequent cardiac diseases in adult cattle [1,2,3,4,5]. This condition is commonly reported as a consequence of infection of the endocardial surfaces due to a persistent bacteriemia [1,6]. Bacterial endocarditis occurs most commonly on the atrioventricular valves and pulmonic valve, and rarely involves the aortic valve in cattle [7,8,9]. Occasionally, mural endocardium adjacent to the valves can also be affected [4,10]. 

Clinical signs of bacterial endocarditis in cattle are variable and frequently insidious because heart failure is only present in the advanced stages of the disease [11]. A heart murmur secondary to valvular insufficiency can be auscultated in cows affected by bacterial endocarditis [2,10], but similar findings can also be heard in cases of congenital and acquired heart defects (CHD) [2] or in apparently healthy cows [12].

Congenital heart diseases as ventricular septal defect (VSD) can facilitate bacterial endocarditis as a consequence of turbulent blood flow through the defect, causing damage to the endocardium; these changes can facilitate bacterial colonization on the valvular and mural endothelium [13]. The risk of secondary bacterial endocarditis in patients affected by VSD is reported in humans, dogs and horses [14,15,16].

To the authors’ knowledge, endocarditis with a VSD has been reported in cattle only in one case report, in which the CHD had been previously diagnosed [17]. The aim of this case report is to describe the clinical presentation and the electrocardiographic, echocardiographic and post-mortem findings in an adult cow affected by mural endocarditis with a small VSD that had not been previously diagnosed. 

## 2. Case Presentation

**Figure 1:**
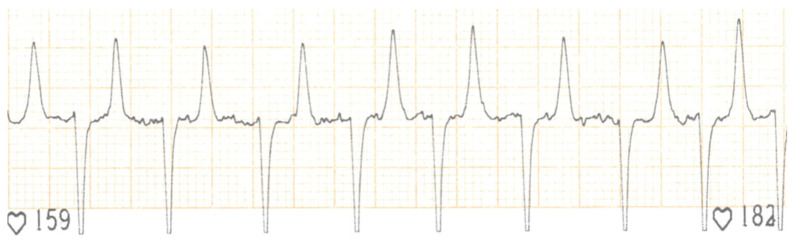
Electrocardiographic tracing (base-apex lead, paper speed 25 mm/s, amplitude 10 mm/mV). P waves are absent, the QRS morphology and duration are normal (reference interval for duration is 70 ms with a range of 50–130 ms) but RR intervals are irregular and f waves with a variable morphology are present. Heart rate is 159–182 bpm.

An eight-year-old female Holstein Friesian cow was examined for an arrhythmia at a dairy farm of Umbria (Central Italy). Ten days prior to the examination, the cow presented dysorexia and a sudden decreased in milk production. The referring veterinarian had revealed hyperthermia (39.5 °C) and arrhythmia; therefore, the cow had been treated with ampicillin/dicloxacillin (5 mL/100 kg, once a day intramuscularly, for 3 days; Cloxalene plus, Fatro, Ozzano dell’Emilia, Italy) and ketoprofen (3 mL/10 kg, once a day intramuscularly, for 3 days; Vetketofen, Merial, Assago, Italy). After the medical treatment, the cow showed weakness, a rectal temperature of 38.7 °C, jugular pulses and pale mucous membranes. Tachycardia with irregular rhythm (150 beats per minute) and tachypnea (40 breaths per minute) with harsh bronchovesicular sounds and no evidence of cardiac murmurs were also revealed. Being clinical signs consistent with a cardiac disease (myocarditis, pericarditis, endocarditis or neoplasm) and congestive heart failure, an electrocardiogram and echocardiographic examination were performed. The electrocardiogram showed an absence of P waves, irregular RR interval, normal QRS morphology and undulation of the baseline (‘f’ waves). These electrocardiographic findings were suggestive of atrial fibrillation with high ventricular response rate (Figure 1). 

**Figure 2:**
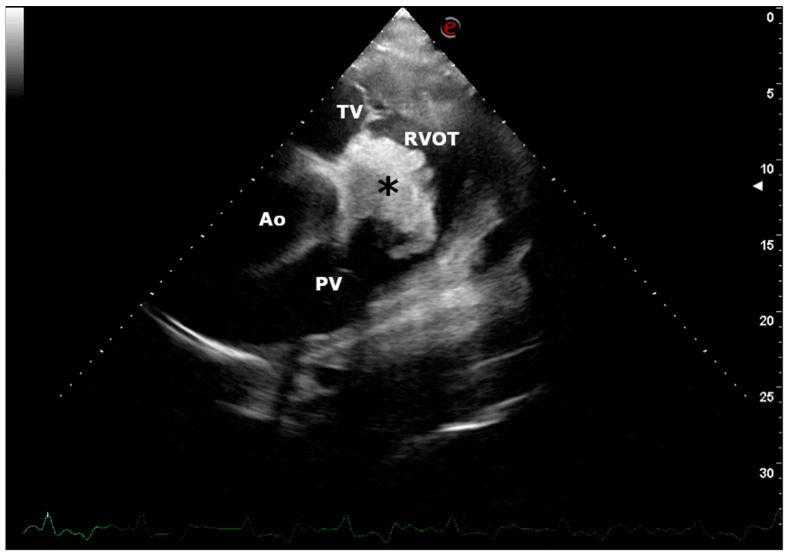
Echocardiographic image. Right parasternal short axis view at the heart base showing a large hyperechoic mural vegetation (⁎) between tricuspid (TV) and pulmonic (PV) valves in the right ventricle outflow tract (RVOT). Ao, aorta; e, orientation marker; scale 5–30, depth setting of the image (cm).

Transthoracic echocardiographic examination was performed using an ultrasound unit equipped with a multifrequency 1–4 MHz phased-array transducer (MyLab^TM^ Omega, Esaote, Genova, Italy). A large vegetation originating from the endocardium between the tricuspid and pulmonic valves in the right ventricle outflow and a mild enlargement of the right ventricle (diastolic right ventricular internal diameter was 3.5 cm; reference interval: mean 2.27 ± 0.76 cm, min-max value 0.78–3.2 cm) were observed (Figure 2). No valvular regurgitation or other turbulent flow were demonstrated by colour-flow Doppler. Clinical, electrocardiographic and echocardiographic findings were suggestive of mural endocarditis (typical proliferative aspect) associated with right congestive heart failure. Moreover, an embolic pneumonia due to thrombi embolized from the vegetative endocarditis lesion into the pulmonary circulation was suspected.

**Figure 3:**
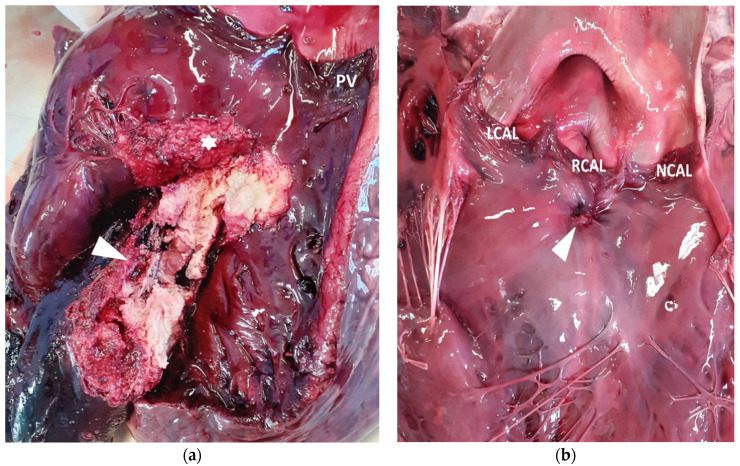
Opened heart. (**a**) Right ventricular view showing a large friable, yellow-to-red mass (arrowhead) adherent to interventricular septum just under the tricuspidal angular leaflet (asterisk); PV: pulmonary valves. (**b**) Left ventricular view showing a small interventricular defect (arrowhead) just below the right coronary aortic leaflet (RCAL); LCAL: left coronary aortic leaflet; NCAL: non coronary aortic leaflet.

Based on the severity of the clinical signs, electrocardiographic and echocardiographic findings and prognosis, the owner elected to slaughter the cow. The day after the clinical evaluation, the cow suddenly died. A field necroscopy was performed. On post-mortem gross examination, a focal, well demarked 3 × 4 cm abscess was present in the chondro-costal junction of the third right rib, oozing thick caseous pus on cut surface. Both lungs had multifocal to coalescing abscesses, yellow to green in colour, with interlobular emphysema. The heart was moderately enlarged, mostly due to right ventricular enlargement; in the right ventricular outflow tract, a large (3 × 4 cm), irregular, friable, yellow mass was present, originating about 2 cm under the pulmonic valve and bulging under the angular leaflet into the inflow tract (Figure 3). In the left ventricle, a small (3 × 4 mm) muscular septal defect was evident under the right coronary cusp of the aortic valve, partially covered by a fibrous edge and clots of fibrin (Figure 3). On cut surface, this small VSD matched with the large right-sided mass that partially obliterated it, even if a narrow tract was evident using a probe. The mass was adherent to the inferior aspect of the tricuspidal angular leaflet. All the other tricuspidal and mitral leaflets were normal. The inspection of the abdominal viscera was limited to liver and kidneys, and both were normal.

Histologically, the mass was composed of bright eosinophilic amorphous material (fibrin) in which nuclear debris and multifocal colonies of small coccoid bacteria were embedded; marginally, the lesion was bordered by a connective tissue ranging from immature and well vascularized (granulation tissue) to well differentiated fibrous tissue. All the other tissues examined were unremarkable.

Culture from the abscess and thrombus was performed. Both samples were cultured on agar plates supplemented with 5% defibrinated sheep blood and MacConkey agar e Mannitol salt agar (Liofilchem, Teramo, Italy) under aerobic and microaerobic conditions for 24–48 h at 37 °C. Phenotypic characterizations were performed by conventional biochemical assay and the API-Coryne system (Biomérieux, Craponne, France) in accordance with the manufacturer’s instructions. *Trueperella pyogenes* was isolated in pure culture.

## 3. Discussion

Congenital heart defects are rare in cattle, a prevalence of 0.17% being reported in two large necropsy studies [18,19]. Most frequently encountered CHD is represented by VSD, although complex CHD, such as tetralogy of Fallot, transposition of the great vessels and double outlet right ventricle, are occasionally reported in this species [18,20,21,22,23,24]. In cattle, a diagnosis of CHD is suspected when clinical signs such as respiratory distress, weakness and heart murmur are noted [5,11,20,25]. Additionally, a history of failure to thrive and/or respiratory disease unresponsive to appropriate therapy can be reported [25,26]. Prognosis in cattle affected by CHD is guarded to poor, being associated with stunted growth and poor productive performance, as well as sudden death [5,27]. In the cow of this report, VSD went unnoticed as no clinical signs suggestive of CHF, such as stunted growth, poor productive performance or a loud heart murmur, were noted. This is not surprising because cows with minor VSD can remain asymptomatic and have a normal productive life for a long period [4,25].

Bacterial endocarditis is associated with CHD in humans, dogs and horses [14,15,16]. The endocardium can be damaged by turbulent blood flow due to the defect and becomes more susceptible to bacterial colonization if persistent bacteremia occurs [1,6]. Bacterial endocarditis occurs most commonly on the valve leaflets but, occasionally, mural endocardium adjacent to the valves can also be affected [4,7,8,9,10]. In this case report, the pathological findings suggest that the turbulent blood flow through the VSD, causing damage to the endocardium, facilitated mural endocarditis, because valvular leaflets were not involved. As reported in the literature, a primary chronic active source of infection such as mastitis, metritis, arthritis or liver abscesses is essential to the pathophysiology of the bacterial endocarditis [1,7,28]. Chronic mastitis, chronic metritis, traumatic reticuloperitonitis, musculoskeletal abscess formation and cellulitis are potential sources of infection [29]. Similarly, in the cow of this report, bacterial colonization on the mural endothelium could occur as a consequence of bacteriemia secondary to the costal abscess seen during the necroscopy. Although this abscess had been previously unnoticed by the owner, we suppose this to be secondary to a trauma. 

The most common cause of bacterial endocarditis are *Trueperella pyogenes* and *Streptococcus* spp. [30]; in this case, *Trueperella* was cultured by cardiac thrombus and costal abscess, confirming the embolic pathogenesis of mural endocarditis.

*Trueperella pyogenes*, previously classified as *Arcanobacterium pyogenes* and *Actinomyces pyogenes*, is a Gram-positive, pleomorphic, non-motile, non-spore-forming rod belonging to the family *Actinomycetaceae*. This microorganism is an opportunistic pathogen which is considered to be part of the mucosal biota of the gastrointestinal, upper respiratory or urogenital tracts of animals. *Trueperella pyogenes* infections are most common in cattle, sheep and pigs, but are also able to cause disease in a large number of animal species including deer, elephants, chickens, horses, dogs, cats, bison and camels. *Trueperella pyogenes* is responsible for purulent infections such as abscesses, metritis, mastitis and pneumonia, which could cause a reduction in production and reproductive performance with economic losses in livestock breeding. The clinical course of these infections could be severe, especially in the case of misdiagnosis or inappropriate antibiotic treatment.

Early clinical signs of bovine bacterial endocarditis are not frequently observed, and the prognosis is reported to be guarded to poor [3,11]. A heart murmur secondary to valvular insufficiency can be auscultated in cows affected by endocarditis of the cardiac valves [2,10]. In the case of this report, no murmur was auscultated because no valvular insufficiency was present. Moreover, the VSD was partially occluded by the endocardial vegetation and no murmur can be heard. These clinical findings were confirmed by Doppler echocardiographic examination. 

Echocardiography is a safe and non-invasive imaging technique that can be useful to confirm or rule out the presence of cardiac disease in cattle [5,25,26]. Moreover, this diagnostic tool is readily available and can be performed in veterinary hospitals as well as in farms. In the cow of this report, echocardiography allowed us to obtain the presumptive diagnosis of mural endocarditis at the farm, although the presence of a small VSD was not observed. The authors believe that the failing in the echocardiographic diagnosis of VSD was due to the absence of an evident blood flow thought the defect. B-dimensional echocardiography can fail in the visualization of small VSD, and the turbulent flow visualized using the Doppler modality can be useful for a diagnosis *in vitam*.

Electrocardiographic examination is routinely performed by a simple base apex lead system in cattle and is mainly used for the assessment of arrhythmias [5]. In the cow of this report, an arrhythmia was detected at the physical examination, and the electrocardiogram performed in farm allowed the diagnosis of an atrial fibrillation. Atrial fibrillation has been frequently reported in large animals, such as horses; however, few reports of its occurrence in cattle are present in the literature. A variety of cardiac diseases, such as valvular endocarditis [31,32,33] or traumatic pericarditis [32,33], have been reported as the cause of atrial fibrillation in cattle.

To the authors’ knowledge, endocarditis associated with a VSD in adult cows has been previously reported only in one case report [17]. The authors of this report described a case of vegetative endocarditis involving an aortic cusp, the tricuspid valve, and the endocardial site of a subaortic perimembranous VSD, previously diagnosed. This cow also showed a disseminated intra vascular coagulopathy and lameness secondary to arterial thromboembolism [17]. In our case report, the VSD was localized in the subaortic region, but the vegetative endocarditis lesions extended in the right ventricle outflow tract. The embolic pneumonia found at the necroscopy could have been due to thrombi shed from the vegetative endocarditis lesions in the right ventricle outflow tract which, once embolized into the pulmonary circulation, became lodged in the distal arteries of the lungs. This is already reported in animals affected by valvular endocarditis, in which fragments of these lesions can embolize in musculoskeletal system, lungs, spleen or liver [28]. Infective endocarditis frequently results in embolization, leading to metastatic infection and organ infarction [28].

## 4. Conclusions

This case report describes a rare consequence of a small VSD that had not been previously diagnosed in an adult cow. Vegetative endocarditis superimposed on VSD resulted in pulmonary embolization and metastatic pneumonia. The clinical and pathological findings presented in this report suggest that VSD can predispose to mural endocarditis and should be considered in cows affected by this commonly acquired cardiac disease with a primary chronic and active source of infection.

## Data Availability

The data presented in this study are available in the article.
